# Supplementary material for: Activation of Metabotropic Glutamate Receptor (mGlu2) and Muscarinic Receptors (M1, M4, and M5), Alone or in Combination, and Its Impact on the Acquisition and Retention of Learning in the Morris Water Maze, NMDA Expression and cGMP Synthesis
Source: Biomolecules. 2023 Jun 30;13(7):1064. doi: 10.3390/biom13071064 (PMC10377483; doi:10.3390/biom13071064)
Supplement: Supplementary file 1 [file biomolecules-13-01064-s001.zip › biomolecules-2427112-supplementary.pdf]

Figures.

Acquisition Trial

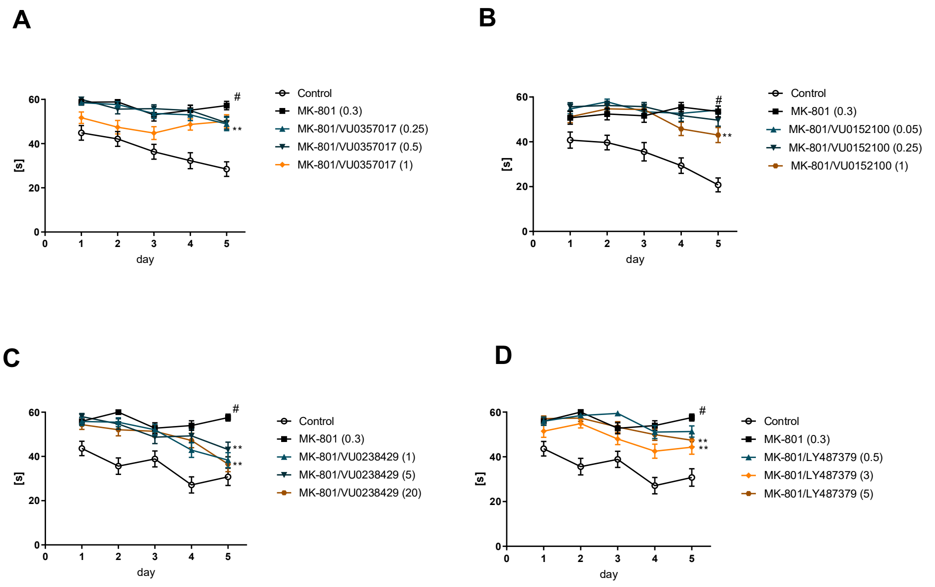

Figure S1. The effects of VU0357017 (A), VU0152100 (B), VU0238429 (C) and LY487379 (D) on MK-801-induced amnestic effect on the acquisition of spatial memory in the MWM in CD1 mice. The values are expressed as the means±SEM. #P<0.001 indicates the difference MK-801-treated mice vs. the respective vehicle (at least N=10) and ** P<0.05 indicates the difference between MK-801 group and the treatment groups.

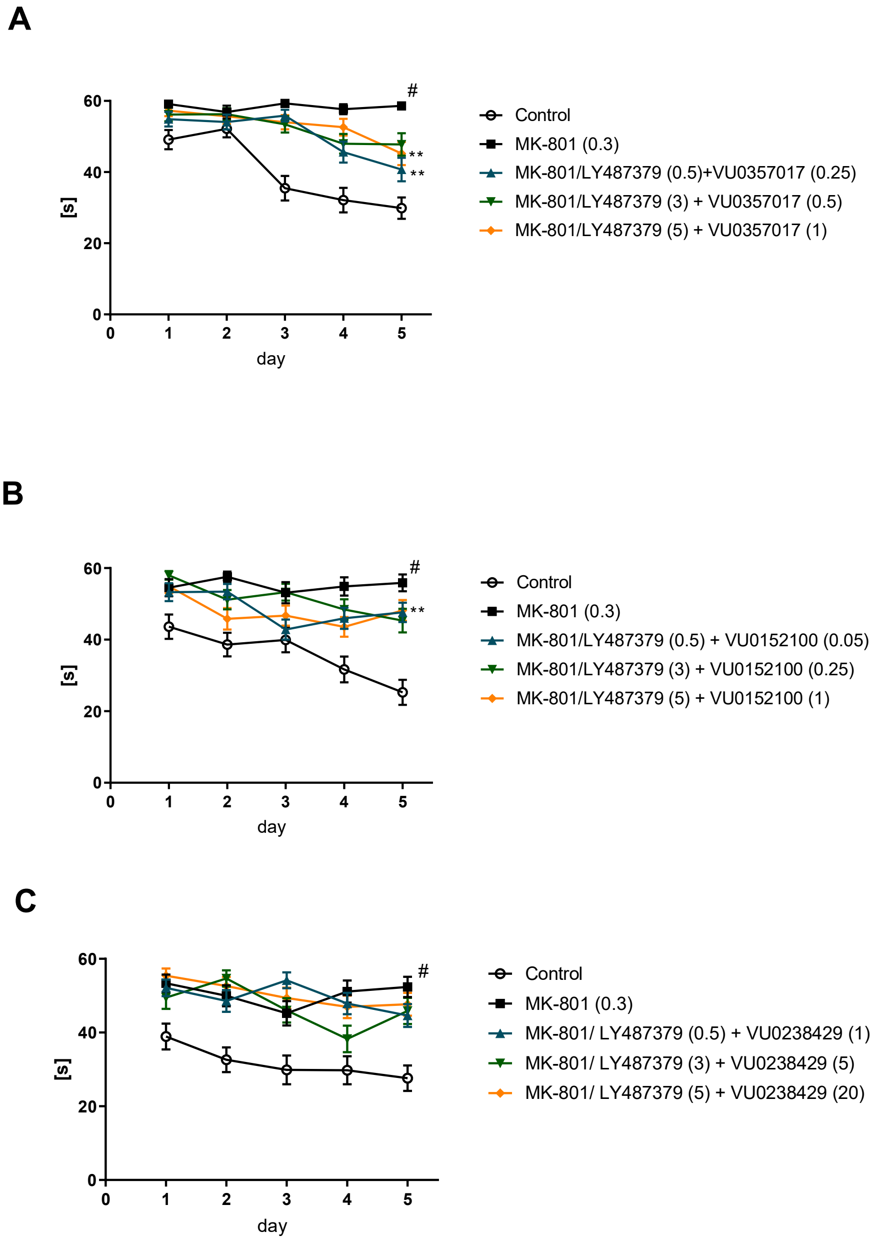

Figure S2. The effects of concomitant administration of LY487379 with VU0357017 (A), LY487379 with VU0152100 (B), and LY487379 with VU0238429 (C) on MK-801-induced amnestic effect on the acquisition of spatial memory in the MWM in CD1 mice. The values are expressed as the means±SEM. #P<0.001 indicates the difference MK-801-treated mice vs. the respective vehicle (at east N=10) and ** P<0.05 indicates the difference between MK-801 group and the treatment groups.

Retention trial

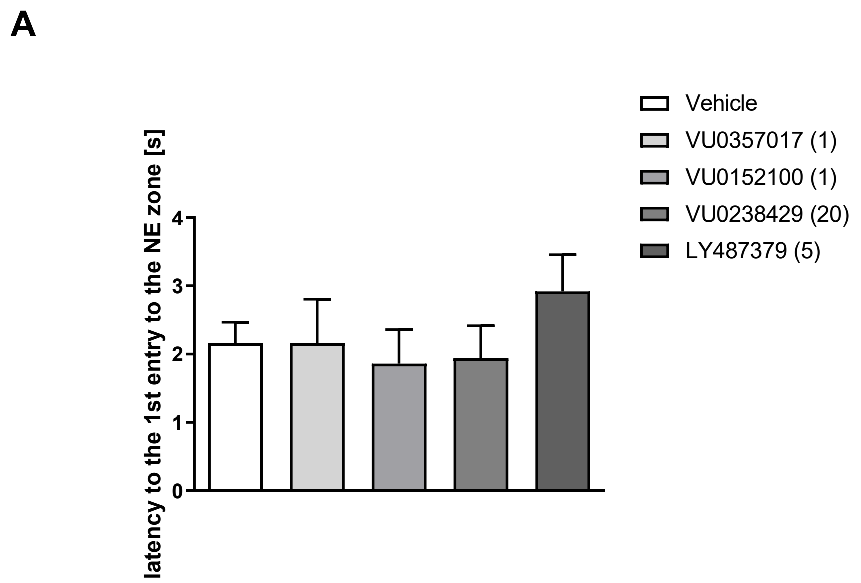

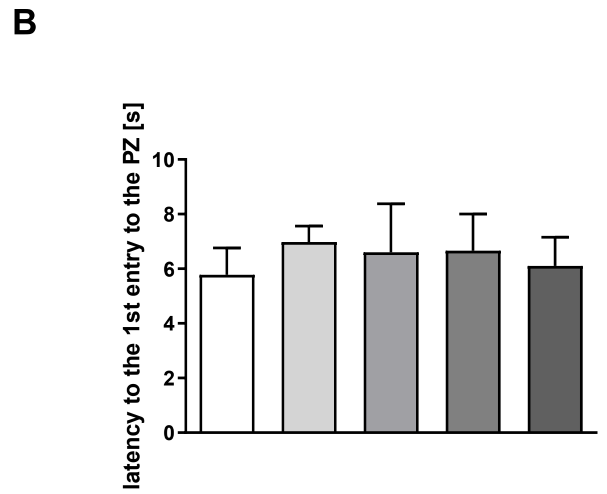

Figure S3. The effects of VU0357017, VU0152100, VU0238429 and LY487379 alone in the retention of spatial memory in the MWM in CD1 mice. The values are expressed as the means±SEM. Doses (mg/kg) are indicated in parentheses. Results are given as the latency to the 1st entry to NE zone (A) and latency to the 1st entry toPZ (B).

Heat maps.

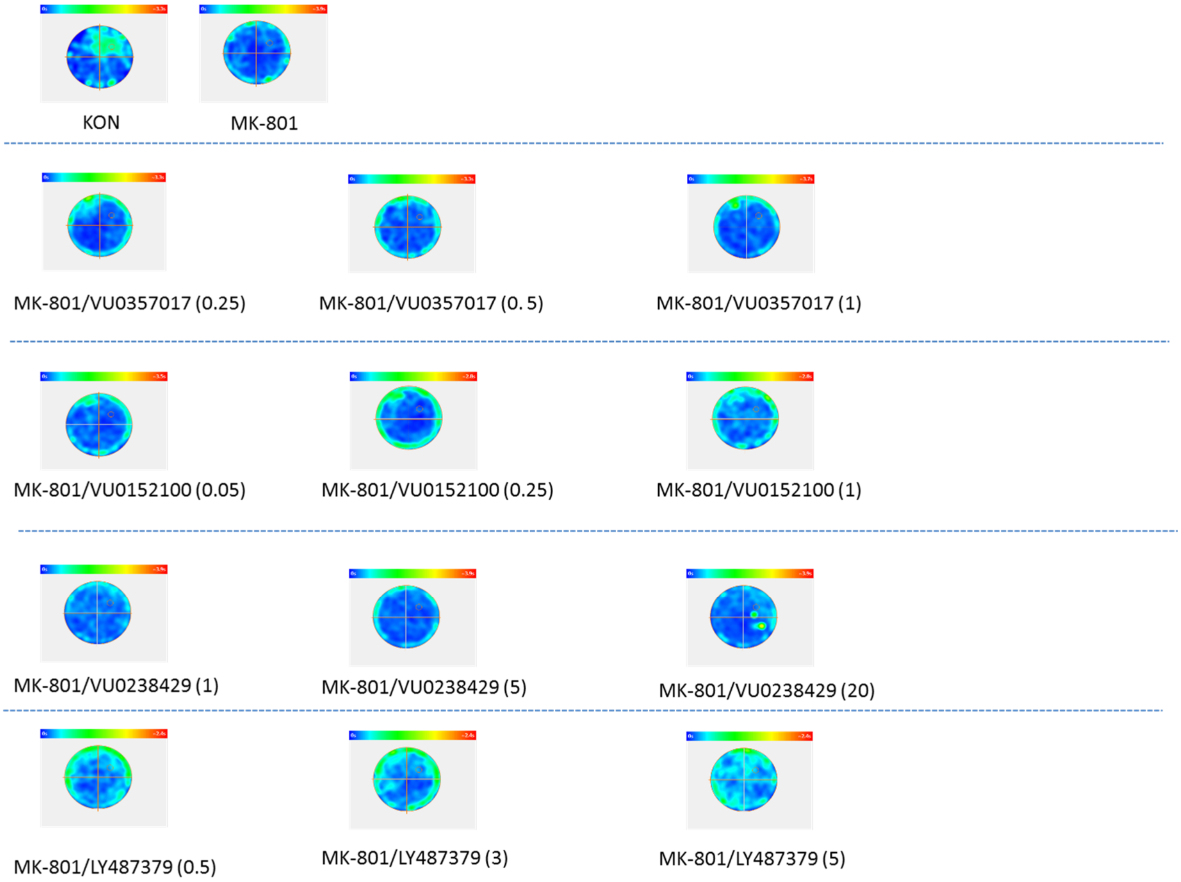

Figure S4. Heat maps reflecting the time spent swimming in particular areas of the maze. The maps show mean data for different treatment groups. Dark blue – no time spent in the area, red – the most time spent in the area. The circle represents platform zone.

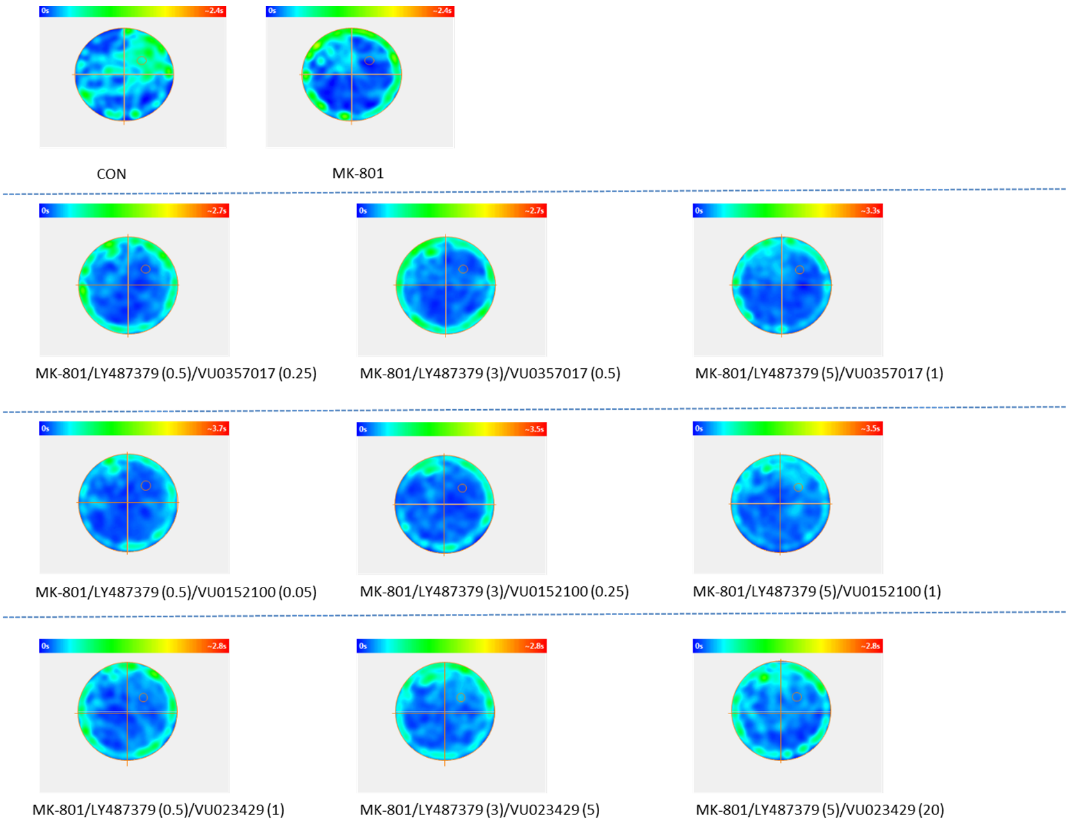

Figure S5. Heat maps reflecting the time spent swimming in particular areas of the maze. The maps show mean data for different treatment groups. Dark blue – no time spent in the area, red – the most time spent in the area. The circle represents platform zone.

Tables

	Average speed [m/s]	
**control**	0	0,219±0,02
**MK-801**	0.3 mg/kg	0,213±0,028
**MK-801+VU0357017**	0.25 mg/kg	0,250±0,029
	0.5 mg/kg	0,231±0,042
	1 mg/kg	0,240±0,039
**MK-801+VU0152100**	0. 05 mg/kg	0,210±0,034
	0.25 mg/kg	0,239±0,041
	1 mg/kg	0,233±0,029
**MK-801+VU0238429**	1 mg/kg	0,231±0,02
	5 mg/kg	0,245±0,047
	20 mg/kg	0,246±0,071
**MK-801+LY487379**	0.5 mg/kg	0,226±0,032
	3 mg/kg	0,228±0,024
	5 mg/kg	0,241±0,030
**MK-801/LY487379/VU0357017**	0.5+0.25 mg/kg	0,258±0,041
	3+0.5 mg/kg	0,269±0,038
	5+1 mg/kg	0,245±0,031
**MK-801/LY487379/VU0152100**	0.5+0.05 mg/kg	0,248±0,02
	3+0.25 mg/kg	0,245±0,016
	5+1 mg/kg	0,237±0,037
**MK-801/LY487379/VU0238429**	0.5+1 mg/kg	0,228±0,040
	3+5 mg/kg	0,259±0,029
	5+20 mg/kg	0,229±0,042

Table S1. Average speed of animals measured during one minute trial during the retention phase. Results are indicated as means ± SEM and indictated as [m/s].

Statistical analysis

	NE-zone related parameters	PZ parameters
Latency to the 1st entry to the zone	t=2.310, df=18, P<0.05	t=2.565, df=12, p<0.05
Number of entries	t=2.172, df=17, p<0.05	t=2.732, df=16, p<0.05
Distance travelled until 1st entry to the zone	t=3.894, df=16, p<0.01	t=3.197, df=8, p<0.05
Path efficiency to the 1st entry to the zone	t=2.55, df=17, p<0.05	t=2.55, df=17, p<0.05
Time in the zone	t=2.35, df=17,p<0.03	*Not measured*
Distance travelled in the zone	*Not measured*	t=2.825, df=18 p<0.05

Table S2. Specification of parameters measured in MWM both in the NE zone and in the PZ (platform zone), that were impaired by MK-801 administration in the retention phase. Statistical analysis were performed with the Student's t-test.

	**VU0357017** (0.25, 0.5, 1 mg/kg)	**VU0152100** (0.05, 0.25, 1 mg/kg)	**VU0238429** (1, 5, 20 mg/kg)	**LY487379** (0.5, 3, 5 mg/kg)
		**NE zone parameters**		
number of entries into the zone	$\mathbf{F_{(3.35)} = 2.968}$ **$p < 0.05$** **1 mg/kg**	$F_{(3.36)} = 1.489$ $p = 0.2339$	$\mathbf{F_{(3.33)} = 2.956}$ **$p < 0.05$** **20 mg/kg**	$F_{(3.35)} = 1.077$ $p = 0.3717$
time in the zone	$F_{(3.36)} = 0.9181$ $p = 0.4419$	$F_{(3.35)} = 0.6410$ $p = 0.5938$	$H(3) = 2.457$ $p = 0.4832$	$F_{(3.35)} = 0.3405$ $p = 0.7961$
distance travelled in the zone	n/a	$F_{(3.36)} = 0.9426$ $p = 0.4302$	n/a	n/a
distance travelled until 1st entry into the zone	n/a	**$H(3) = 8.393$** **$p < 0.05$** **0.5, 1 mg/kg**	**$H(3) = 8.273$** **$p < 0.05$** **20 mg/kg**	$\mathbf{F_{(3.35)} = 3.554}$ **$p < 0.05$** **5 mg/kg**
latency to 1st entry into the zone	n/a	**$H(3) = 12.11$** **$p < 0.01$** **0.05, 0.5, 1 mg/kg**	**$H(3) = 7.905$** **$p < 0.05$** **5, 20 mg/kg**	**$H(3) = 9.159$** **$p < 0.05$** **5 mg/kg**
path efficiency to 1st entry into the zone	n/a	$H(3) = 5.400$ $p = 0.1448$	$H(3) = 2.665$ $p = 0.4462$	$\mathbf{F_{(3.33)} = 3.282}$ **$p < 0.05$** **5 mg/kg**
		**PZ parameters**		
number of entries into the zone	$H(3) = 1.327$ $p = 0.7226$	$H(3) = 2.029$ $p = 0.5665$	$H(3) = 2.307$ $p = 0.5112$	$H(3) = 3.597$ $p = 0.3083$
time in the zone	$H(3) = 1.019$ $p = 0.6007$	$H(3) = 3.125$ $p = 0.3727$	$H(3) = 2.166$ $p = 0.5387$	**$H(3) = 8.282$** **$p < 0.05$** **0.5, 5 mg/kg**
distance travelled until 1st entry into the zone	**$H(3) = 9.653$** **$p < 0.05$** **0.5, 1 mg/kg**	n/a	n/a	n/a
latency to 1st entry into the zone	**$H(3) = 0.0217$** **$p < 0.05$** **0.5, 1 mg/kg**	$H(3) = 3.603$ $p = 0.3077$	$H(3) = 6.900$ $p = 0.0751$	$\mathbf{F_{(3.23)} = 3.575}$ **$p < 0.05$** **3, 5 mg/kg**
path efficiency to 1st entry into the zone	**$H(3) = 12.13$** **$p < 0.01$** **0.5, 1 mg/kg**	n/a	n/a	n/a

Table S3. Statistical analysis of the results obtained in the retention phase of MWM for individual compounds. Investigated doses are indicated in parentheses. n/a—not applicable.

Treatment	Structure	ANOVA results	Tukey's post hoc comparison	
			MK-801 vs. MK-801/treatment	Control vs. MK-801/treatment
**VU0357017**	FC	$F_{(2.20)} = 29.29$, $p < 0.0001$	$p < 0.0001$	$p < 0.007$
	Hipp	$F_{(2.21)} = 25.19$, $p < 0.0001$	$p < 0.0001$	$p < 0.002$
**VU0152100**	FC	$F_{(2.20)} = 30.39$, $p < 0.0001$	$p < 0.0001$	$p < 0.004$
	Hipp	$F_{(2.21)} = 49.40$, $p < 0.0001$	$p < 0.0001$	$p < 0.0001$
**VU0238429**	FC	$F_{(2.20)} = 25.82$,$p < 0.0001$	$p < 0.0001$	$p < 0.04$
	Hipp	$F_{(2.21)} = 24.97$, $p < 0.0001$	$p < 0.0001$	$p < 0.0003$
**LY487379**	FC	$F_{(2.20)} = 38.76$, $p < 0.0001$	$p < 0.0001$	$p < 0.0006$
	Hipp	$F_{(2.20)} = 25.84$, $p < 0.0001$	$p < 0.0001$	$p < 0.0003$

Table S4. Statistical analysis of the cGMP levels after administration of individual compounds at highest doses. FC—frontal cortex, Hipp—hippocampus.
